# Structural Basis of Gate-DNA Breakage and Resealing by Type II Topoisomerases

**DOI:** 10.1371/journal.pone.0011338

**Published:** 2010-06-28

**Authors:** Ivan Laponogov, Xiao-Su Pan, Dennis A. Veselkov, Katherine E. McAuley, L. Mark Fisher, Mark R. Sanderson

**Affiliations:** 1 Randall Division of Cell and Molecular Biophysics, King's College London, London, United Kingdom; 2 Division of Basic Medical Sciences, St George's University of London, London, United Kingdom; 3 Diamond Light Source, Didcot, United Kingdom; Tulane University Health Sciences Center, United States of America

## Abstract

Type II DNA topoisomerases are ubiquitous enzymes with essential functions in DNA replication, recombination and transcription. They change DNA topology by forming a transient covalent cleavage complex with a gate-DNA duplex that allows transport of a second duplex though the gate. Despite its biological importance and targeting by anticancer and antibacterial drugs, cleavage complex formation and reversal is not understood for any type II enzyme. To address the mechanism, we have used X-ray crystallography to study sequential states in the formation and reversal of a DNA cleavage complex by topoisomerase IV from *Streptococcus pneumoniae*, the bacterial type II enzyme involved in chromosome segregation. A high resolution structure of the complex captured by a novel antibacterial dione reveals two drug molecules intercalated at a cleaved B-form DNA gate and anchored by drug-specific protein contacts. Dione release generated drug-free cleaved and resealed DNA complexes in which the DNA gate instead adopts an unusual A/B-form helical conformation with a Mg^2+^ ion repositioned to coordinate each scissile phosphodiester group and promote reversible cleavage by active-site tyrosines. These structures, the first for putative reaction intermediates of a type II topoisomerase, suggest how a type II enzyme reseals DNA during its normal reaction cycle and illuminate aspects of drug arrest important for the development of new topoisomerase-targeting therapeutics.

## Introduction

The bacterial type II enzymes topoisomerase IV and gyrase have important roles in DNA replication [1]–[4]. Topoisomerase IV, a tetramer of two ParC and two ParE subunits, unlinks daughter chromosomes prior to cell division whereas the related enzyme gyrase, a GyrA_2_GyrB_2_ tetramer, supercoils DNA and helps unwind DNA at replication forks [3]. Both enzymes act *via* a double-strand DNA break involving a cleavage complex (Figure S1 and Movie S1). Detailed understanding of their reaction pathway has been frustrated by the transient nature of this key intermediate and the absence of relevant drug-free structures. Antibacterial fluoroquinolones stabilize the cleavage complex in a Mg^2+^-dependent fashion facilitating biochemical and structural work [3], [5]. Recent studies have also identified quinazolinediones as a new class of topoisomerase IV inhibitors with activity against *Streptococcus pneumoniae* and other Gram-positive pathogens [6]–[8]. Here we use a novel quinazolinedione to generate crystals of *Streptococcus pneumoniae* topoisomerase IV captured as a cleavage complex, and by exploiting the slow resealing of the complex [9], we have now obtained the first diffracting crystals of sequential catalytically-competent drug-free-DNA cleaved and resealed complexes. The structures of these complexes explain unique dione activities (such as lack of cross-resistance with quinolones [9]–[11]) and provide a structural basis for reversible DNA strand breakage by a type II topoisomerase.

## Results and Discussion

**Figure 1:**
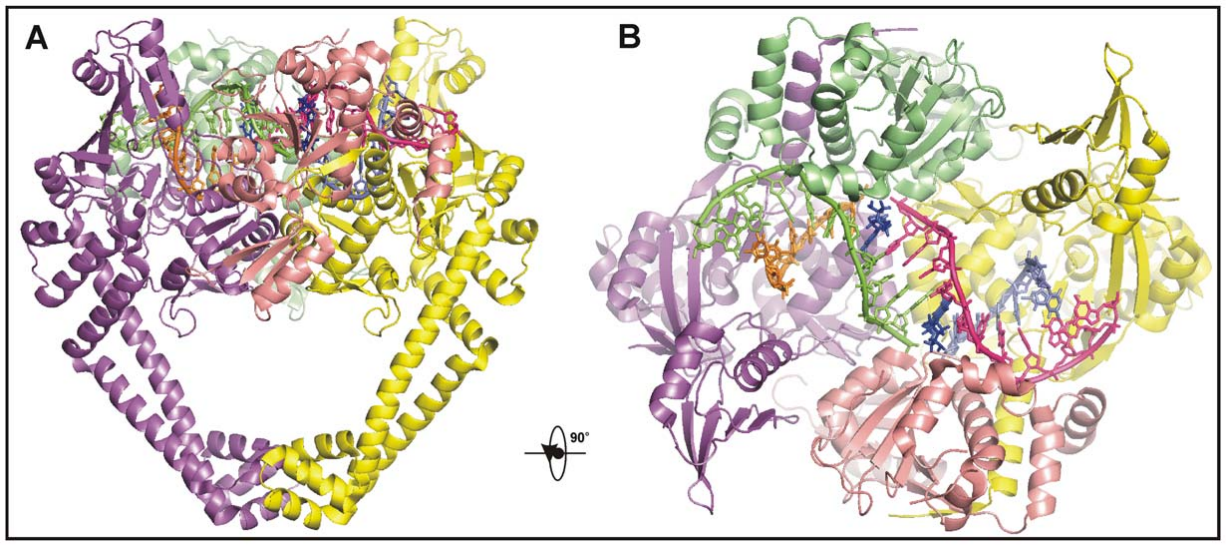
Architecture of a dione-arrested DNA complex of topoisomerase IV. **A–B**, Orthogonal views of the topo IV-DNA complex stabilized by PD 0305970. ParC is in purple/yellow, ParE is in salad green/light rose, DNA strands are in pink, blue, orange and light-green, PD 0305970 is in dark blue.

**Figure 2:**
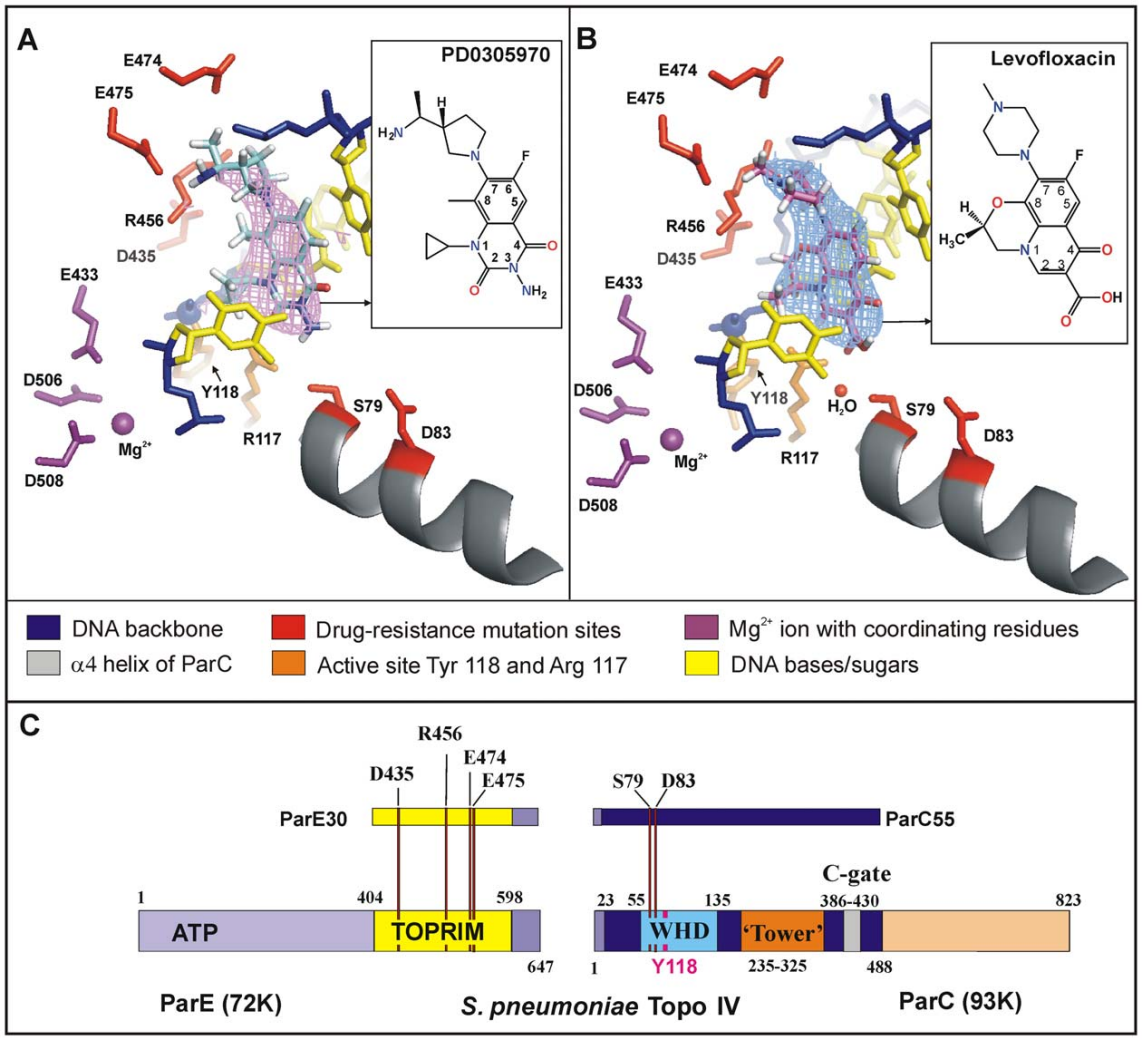
Unique interactions govern topoisomerase IV arrest on DNA. **A** and **B**, Cartoon/stick representation of the drug-binding pockets of topoisomerase IV-DNA in complex with PD 0305970 and levofloxacin, respectively. The σA-weighted 2F_obs_-F_calc_ map is contoured around PD 0305970 (pink) and levofloxacin (light-blue) at 1.5σ. **C**, Domain organization of topo IV from *S. pneumoniae* with individual sub-regions highlighted by individual colours. Resistance mutation sites within the topoisomerase sequence are indicated by red lines.

We co-crystallized the potent dione PD 0305970 with the *S. pneumoniae* ParC breakage-reunion domain (ParC55, residues 1–488) and ParE TOPRIM domain (ParE30, residues 404–647) in the presence of a 34-bp DNA duplex (the E-site) [12] (Figures 1 and 2). The X-ray crystal structure of the complex was determined to 3.1 Å revealing a closed ParC55 dimer flanked by two ParE30 monomers (Figure 1). In contrast to a yeast topoisomerase II core domain in complex with an artificially-nicked surrogate DNA substrate [13], the C-terminal C-gate region (comprising helices α14, α18 and α19) is closed in the dione structure as it is in lower resolution quinolone structures [5]. The G-segment DNA is bound across the ParC dimer interface in a ‘U shape’ in which the central 18-bp region is well resolved. This region carries a 4-bp staggered DNA break in which the two active site ParC Tyr 118 residues are linked covalently to the 5′ phosphate ends of the break. Two dione molecules are intercalated in the space between the +1 and –1 nucleotides at the two ends of the staggered DNA cut. These features confirm the capture of a dione-stabilized complex, the first high resolution structure of a cleavage complex reported for a type II topoisomerase.

The structure reveals the disposition of protein side-chains, magnesium ions and DNA that form the drug binding pockets (Figure 2A and Figure S2A). The dione sits on the purine base at +1 with the electronegative fluorine at C-6 (optimal for drug activity) interacting with partially charged atoms in the DNA bases (Figure S2A). We interpret additional density close to the non-scissile phosphodiester linking −1 and −2 nucleotides as a Mg^2+^ ion, consistent with its complexation by Asp 506 and Asp 508, two conserved metal binding residues of the TOPRIM domain [4]. The dione C-2 oxygen makes a key hydrogen-bonded contact with the side-chain of Arg 117, a conserved active-site residue from ParC. The Ser 79 and Asp 83 residues of ParC that are mutated in quinolone resistance [10], [11] are well removed from the dione N-3 amino group, whereas the conserved ParE Arg 456, Glu 474, Glu 475 and Asp 435 residues altered in dione resistance [9] are clustered around the dione C-7 group. Overall, the structure plausibly accounts for the known antimicrobial properties of the diones [9].

Release of dione could be demonstrated by sequential desoaking/soaking with fluoroquinolones affording the levofloxacin cleavage complex with topo IV which we solved at 2.9 Å. The overall architecture of the dione and levofloxacin complexes appears similar, but in fact the drugs are bound differently (Figure 2 and Figure S2). Thus, the ParC residues Ser 79 (mutated in quinolone resistance) and Arg 117 are both coordinated to the levofloxacin C-3 carboxyl through a highly mobile hydrogen-bonded water molecule (absent in the dione structure). Disruption of these contacts in Ser 79 mutants may destabilise drug binding leading to quinolone resistance. Conversely, the bound quinolone does not interact with ParE Glu 474 and Glu 475 residues mutated in dione resistance (Figure S2). The engagement of different protein side-chains accounts for the differential activities and lack of cross-resistance between the dione and quinolone classes of antimicrobial agents [9].

**Figure 3:**
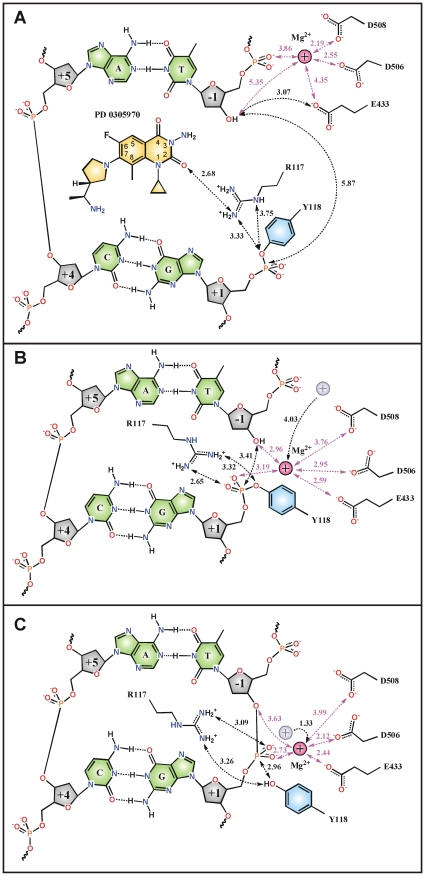
Topoisomerase IV active site with cleaved and resealed DNA. **A–C**, Repositioning of Mg^2+^, DNA and active-site amino acids (Arg 117 and Tyr 118 from ParC; Glu 433, Asp 506 and Asp 508 from ParE) in sequential drug-stabilized (**A**), drug-free cleaved (**B**) and drug-free re-sealed (**C**) topo IV-DNA cleavage complexes, respectively. Current and previous positions of the magnesium ion are shown in light-purple and grey correspondingly. Close intermolecular contacts are indicated by dotted lines.

**Figure 4:**
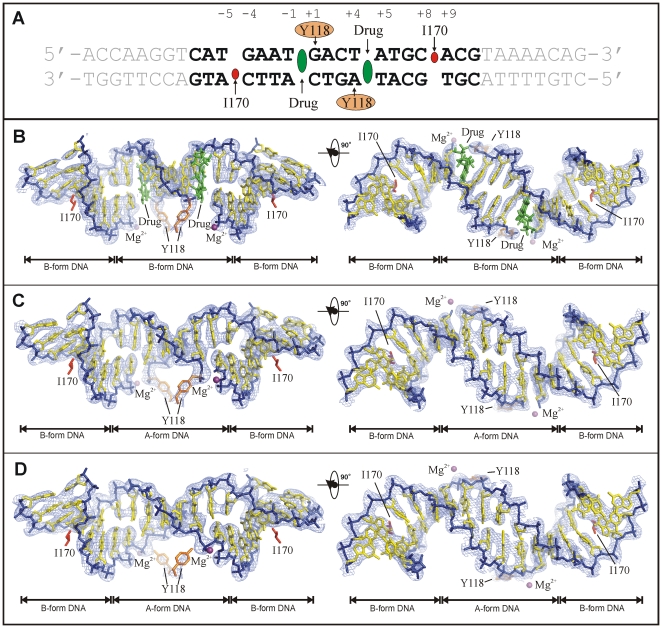
Molecular and conformational changes at the DNA gate. **A**, E-site DNA sequence with important positions involved in protein-DNA and PD 0305970-DNA interactions indicated. The highlighted 18-bp region is well resolved in the crystal structures. **B–D**, Drug-stabilised, drug-free cleaved and drug-free re-sealed topo IV-DNA cleavage complexes respectively. The DNA is in blue/yellow for backbone/bases. Active site tyrosines are in orange, intercalating isoleucines are in red and magnesium ions are in purple. The σA-weighted 2F_obs_-F_calc_ map is contoured around the DNA/Tyr 118 of the structure at 1.5σ (light-blue). In **B** the PD 0305970 molecules are in green. The remaining parts of the complex are omitted for clarity.

Slow resealing of dione cleavage complexes [9] led us to prepare crystals of the dione complex that had been soaked sequentially with EDTA and then with MgCl_2_. The respective structures solved at 3.3 Å and 3.5 Å revealed that in each case the dione had been released, but remarkably the DNA remained cleaved after EDTA and had become resealed following further incubation in the presence of Mg^2+^. These structures represent potential cleavage (Figure 3B) and pre-cleavage (Figure 3C) reaction intermediates and thus provide new insight on events at the gate-DNA notably the role of Mg^2+^ (Figure 3) and DNA conformation (Figure 4). Consistent with this idea, in the sealed DNA complex (Figure 3C), the OH group of Tyr 118 is poised to undergo nucleophilic attack on the phosphorus atom of the phosphodiester group linking +1 and –1 nucleotides potentiated by the positively-charged side-chain of the highly conserved Arg 117. Furthermore, a Mg^2+^ ion now coordinated by Glu 433 and Asp 506 is bound to bridging and apical oxygens of the scissile phosphodiester moiety facilitating tyrosyl attack by stabilizing both the incipient 3′-OH leaving group and tyrosyl phosphate group, as seen in the cleaved complex (Figure 3B). Thus, the Mg^2+^ ions have been repositioned to occupy new sites at which they can promote catalysis at scissile phosphates, a major difference with drug-bound structures (Figure 3A and Figure S2).

Recent biochemical studies have led to a two-metal ion model for DNA cleavage by gyrase and topoisomerase II, as seen for some other (though not all) phosphodiester processing enzymes [4], [14]–[16]. One Mg^2+^ is suggested to bind the 3′ bridging oxygen of the scissile phosphodiester bond stabilizing the 3′ OH leaving group, with a second ion potentiating the catalytic tyrosine OH [4], [15], [16]. Interestingly, in our structures of catalytically competent cleaved and resealed DNA states, a single ion at each scissile DNA site coordinates both sets of reactive DNA groups. Although we cannot exclude the participation of a second more weakly bound Mg^2+^ perhaps stabilised somehow in the holoenzyme, we see no evidence for it in our crystal structures suggesting the possibility that, at least for topoisomerase IV, a single ‘dynamic’ Mg^2+^ is sufficient for transphosphorylation. Similar considerations may hold for the TOPRIM domain of type I topoisomerases that act through a single-strand DNA break [4].

Access to dione-stabilized and drug-free enzyme complexes greatly enhances our understanding of the G-gate and its arrest by drugs (Figure S2, Figure 3 and Figure 4). Drug intercalation critically affects the disposition of catalytic groups within the complex, tying up the active site Arg 117, pushing the reactive 3′ OH and 5′ phosphate DNA ends far apart (5.9 Å) and displacing Mg^2+^ ions by 4 Å (Figure 3A and Figure S2A). Moreover, DNA analysis using the program 3DNA [17], [18] reveals major differences in DNA conformation at the gate (Figure 4). Thus, the drug-free and drug-stabilised complexes all bind the G-DNA in a U-shaped fashion facilitated by the symmetrical DNA insertion of the side-chains of ParC Ile 170 residues between nucleotides +8 and +9 on each strand and flanking the DNA cleavage sites (Figure 4A). The middle 8 nucleotides (−2 to +6) encompassing the cleavage sites adopt the A-helix conformation in the drug-free complexes (Figure 4C, D) but B-form DNA in the drug-stabilised complexes (Figure 4B), whereas the outer regions of the G-DNA are B-form in all cases (Figure 4B–D). The backbone of the G-DNA is trapped within the DNA-binding groove of the protein by both van der Waals and electrostatic interaction (Figure S3A). Basic residues together with the active site magnesium ion run along the DNA backbone, while the neutral side-chain of ParC Ile 170 intercalates into the minor groove. The DNA conformation is further stabilised by the α4 helix intercalated into the major groove. The G-segment is forced into the stretched and unwound A-form conformation (Figure 3B,C) suggesting that A-form DNA may be required for proper gate alignment, cleavage and separation prior to DNA transport. The role of Mg^2+^ would be to oppose separation and promote DNA religation by coordinating the 5′ and 3′ ends of the G-segment. By displacing Mg^2+^ closer to the −1 backbone phosphate group, dione binding promotes cleavage and may favour ‘relaxation’ of the G-DNA into the B-form conformation. These results provide the first three-dimensional insights on events at the G gate.

Reversible cleavage at a gate-DNA is the salient feature underlying the biological activities of all type II topoisomerases yet the nature of this process has hitherto remained obscure. Our structural analysis of dione- and quinolone-stabilized complexes of topoisomerase IV alongside catalytically competent cleaved and resealed states now provides a detailed picture of the architecture of the G-DNA gate and of the events that occur during DNA breakage and reversal. The work sheds new light on the mechanistic roles of Mg^2+^ and DNA conformation at the G-gate, and elucidates how different classes of antibacterial agents interfere with DNA resealing. These insights should aid the design of new antimicrobial agents able to circumvent resistance through novel interactions at the topoisomerase-DNA interface.

## Materials and Methods

### Proteins and DNA

*S. pneumoniae* ParC55 (residues 1–488) and ParE30 (residues 404–647) were produced as the C-terminally and N-terminally His-tagged proteins by over-expression in *E. coli* and purified as described previously [5], [19]. DNA oligomers (5′-ACCAAGGTCATGAATGACTATGCACGTAAAACAG-3′, 5′-CTGTTTTACGTGCATAGTCATTCATGACCTTGGT-3′) were both synthesized by solid-phase phosphoramidite chemistry and doubly HPLC purified by Metabion, Munich.

### Crystallization

Equimolar amounts of ParC55 and ParE30 were incubated (at 4 mg/ml) with the 34 base-pair DNA binding site fragment (1∶1∶1.2 molar ratio respectively) and quinazolinedione PD 0305970 (30 µg/ml) in buffer A (20 mM Tris-HCl pH 7.0, 200 mM NaCl, 10% glycerol (v/v), 1 mM β-mercaptoethanol, 20 mM MgCl_2_, 0.05% NaN_3_ (w/v)) at room temperature for two days. The mixture then was dialysed overnight against the buffer B (20 mM Tris-HCl pH 7.0, 75 mM NaCl, 10% glycerol (v/v), 1 mM β-mercaptoethanol, 20 mM MgCl_2_, 0.05% NaN_3_ (w/v), 1 µg/ml PD 0305970). Then, PD 0305970 was added to a final concentration of 31 µg/ml and the mixture was further incubated at room temperature overnight. Crystallization was performed both by conventional hanging drop vapour diffusion in 24-well Limbro plates (4+2 µl protein mix/reservoir) and by sitting drop in 96-well MRC crystallization plates (600+300 nl protein mix/reservoir). Crystals formed after 7 days from 50 mM Na cacodylate, pH 6.5, 2.5% Tacsimate™, pH 7.0 (Hampton Research Corp.) [20], 62.5 mM KCl, 7.5 mM MgCl_2_, 4–6% (v/v) isopropanol and continued to grow for another 7 days. The crystals were flash-cooled to 100 K into a cryoprotectant buffer C (50 mM Na cacodylate, pH 6.5, 2.5% Tacsimate™, pH 7.0 (Hampton Research Corp.) [20], 62.5 mM KCl, 7.5 mM MgCl_2_, 1 mM β-mercaptoethanol, 30% (v/v) MPD). The best crystals were used to collect the native PD 0305970 datasets with the best resolution of 3.1 Å obtained at the Diamond synchrotron light source, UK. The remaining crystals were harvested into the cryoprotectant buffer C and subjected to 6 changes of the solution (at least 1∶100 dilution each time) with 1-hour intervals in order to back-soak out PD 0305970 from the crystal lattice/remove it from the solution. Subsequently, crystals were divided into groups and each group was soaked overnight in 300 µl cryoprotectant buffer C containing in addition one of the following: 10 mM EDTA (referred to as EDTA crystals) or 3 mM levofloxacin. The best crystals from these groups were flash-cooled in the nitrogen cryostream (100 K). A few crystals from the EDTA-soaked group were subjected to 6 changes of cryoprotectant buffer C over a period of 8 hours in order to remove EDTA and re-introduce Mg^2+^ ions (later on referred to as EDTA-Mg crystals). The best crystals from this group were also flash-cooled in the nitrogen cryostream and used for data collection at the Diamond synchrotron.

### Data Collection

Crystals were tested in-house for diffraction quality using an Oxford Xcalibur Nova CCD diffractometer and then transported for high-resolution data collection at the Diamond Light Source (Oxford, UK) (beamline I03, wavelength 0.97630 Å) using ADSC quantum 315 detectors. Data were integrated and reduced using HKL2000 [21]. To our surprise, the levofloxacin-soaked crystals exhibited good quality diffraction and a relatively high intensity of diffracted reflections to high angle, exceeding that for one of the best native PD 0305970-containing crystals and all other soaked crystals. The diffraction reached a resolution of 2.9 Å with some anisotropic data extending up to 2.6 Å. The best dataset obtained so far for the native PD 0305970-containing crystal extends up to 3.1 Å. Crystals soaked using other drugs as well as the crystals from which PD 0305970 was back-soaked out, diffracted to a lower resolution and did not show a clear and interpretable drug envelope in the 2F_obs_-F_calc_ maps after refinement. By contrast, EDTA and EDTA-Mg crystals exhibited diffraction patterns of relatively high intensity and the resolution of the data obtained extended up to 3.3 Å and 3.5 Å respectively. The highest resolution dataset (from a levofloxacin-soaked crystal) was used to obtain the structure solution and refinement. The structure was solved by molecular replacement in Phaser [22] using as search models our cleavage complex of topo IV from *S. pneumoniae* with moxifloxacin [5]. Refinement was performed in Phenix [23] using the secondary structure restraints derived from the previously solved complex. Rigid body, simulated annealing, positional and TLS refinement has been performed. The refined model with the drug molecules omitted was used to solve the structures of the native PD 0305970-containing crystals as well as the EDTA and EDTA-Mg crystals. For all these additional structures, rigid body refinement, simulated annealing, positional and TLS refinement were performed. The drug molecules, magnesium ions and water molecules were placed during the last stages of refinement according to the missing electron density in the σA-weighted 2F_obs_-F_calc_ and F_obs_-F_calc_ maps. WinCoot [24] was used for interactive model fitting. The structures were verified using WinCoot and ProCheck [25], [26]. The models had good geometry with 82.5/16.2/1.0 (PD 0305970-stabilised cleavage complex), 86.8/11.8/1.1 (levofloxacin-stabilised cleavage complex), 83.9/13.8/1.6 (drug-free complex with cleaved DNA) and 82.4/15.4/1.4 (drug-free complex with re-sealed DNA) percent of residues in favoured/allowed/generously allowed regions of the Ramachandran plot, respectively, and no more than 0.3–0.7% of residues in disallowed regions. The final data collection refinement statistics are given in Table S1. DNA conformation (A/B-form) was analysed using 3DNA [17], [18]. Figures were prepared using PyMOL [27].

To our surprise, the EDTA-soaked crystals did not lose Mg^2+^ ion as it could still be found in the electron density coordinating several negatively charged residues such as Glu 433, Asp 506 as well as the 5′ phosphate groups. The drug had been completely removed, however, the DNA was still cleaved and covalently attached to the active site tyrosines (i.e., drug-free cleaved complex). A similar situation was observed for the EDTA-Mg crystals although in this case the DNA was re-sealed (i.e., drug-free resealed complex). Since apparently the Mg^2+^ ions were retained throughout the process of drug removal and DNA resealing, the role of EDTA in this process remains unclear and the most plausible explanation is that the in-crystal DNA resealing required a longer time than removing the drug by back-soaking. Slow resealing in solution of topoisomerase IV-dione-DNA cleavage complexes following EDTA addition has been reported previously [9], and was at least 60-fold slower than that seen with levofloxacin (X.-S. Pan and L.M. Fisher, unpublished results).

Atomic coordinates and structure factors have been deposited in the Protein Data Bank under accession numbers 3LTN (complex with PD 0305970), 3K9F (complex with levofloxacin), 3KSA (drug-free cleaved complex) and 3KSB (drug-free resealed complex). Raw diffraction images are available on request. Correspondence and request for materials should be addressed to L.M.F and M.R.S (lfisher@sgul.ac.uk, mark.sanderson@kcl.ac.uk).

‘Note Added In Proof: Our manuscript was submitted on April 29, 2010. Whilst our paper was under review, another study appeared (Schmidt BH *et al*. Nature 2010 May 19 [Epub ahead of print] describing a structure of yeast topo II core domain in complex with Zn^2+^ ions and an artificially nicked phosphorothiolate DNA substrate’. It may be mentioned that the four structures we present here involve topoisomerase IV with natural DNA substrate and Mg^2+^ ions and give information for cleaved and resealed complexes determined both in the absence and presence of dione/quinolone drugs'.

## Supporting Information

Figure S1 Schematic model of the DNA transport mechanism employed by a type II DNA topoisomerase and its inhibition by antibacterial/anticancer drug action. The diagram is built using a model of the full-length topo IV which, in turn, is based on the crystal structure of the drug-free cleavage complex of topo IV from *S. pneumoniae* with cleaved DNA (reported in this paper, 3KSA, covering the N-terminal domain of ParC, C-terminal domain of ParE and bound/cleaved G-segment) as well as the crystal structures of the C-terminal domain of GyrA from *B. burgdorferi* (1SUU)^1^ (for C-terminal domain of ParC) and the N-terminal domain of GyrB from *E. coli* (1EI1) (for N-terminal domain of ParE)^2^. Unbound G-segment and the transported T-segment were generated using WinCoot^3^. N-terminal domain of ParC is shown in blue, C-terminal domain of ParC is in cyan, N-terminal domain of ParE is in purple, C-terminal domain of ParE is in yellow, G-segment is in green and T-segment is in red. ΔL stands for a change in the linking number of the DNA per cycle. An animated version of this scheme is available as the Supporting Movie S1 online.
(2.41 MB TIF)

Figure S2 Key interactions within drug-stabilized cleavage complexes of *S. pneumoniae* topoisomerase IV. The cleavage complexes are stabilized by quinazolinedione PD 0305970 (A) and quinolone levofloxacin (B). The important inter-atomic distances are shown in Ångstroms and indicated by arrows. Magnesium ion is in purple, tyrosine is in cyan, DNA bases are in green, DNA sugars are in grey, PD 0305970 is in yellow, levofloxacin is in ‘rose’ and water molecule is in light pink. Nitrogen, oxygen and phosphorus atoms are in blue, red and orange, respectively.
(1.15 MB TIF)

Figure S3 Specifics of the G-segment binding groove and DNA curvature (stereograms). A, Key elements of the DNA-binding cleft of topo IV from *S. pneumoniae*. Residues within 5 Å distance from the DNA molecule are shown in surface representation. Side-chains of basic/acidic amino acids are in blue/red respectively. Active site tyrosine is in orange, the side-chain of intercalating isoleucine 170 is in green, the magnesium ion is in yellow. The G DNA fragment is shown in cartoon representation (cyan). The rest of the complex is omitted for clarity. B and C, modelled ideal A-form and B-form DNA molecules with curvatures of 27 and 33 Å respectively superposed onto the re-sealed G-segment. Models were prepared using CNS^4^. The figure panels were rendered in VMD^5^.
(4.38 MB TIF)

Table S1 Data collection and refinement statistics and Supporting References.
(0.06 MB DOC)

Movie S1 Animated model of the DNA transport mechanism employed by a type II DNA topoisomerases. The animation is created using a model of the full-length topo IV which, in turn, is based on the crystal structure of the drug-free cleavage complex of topo IV from *S. pneumoniae* with cleaved DNA (reported in this paper, 3KSA, covering N-terminal domain of ParC, C-terminal domain of ParE and bound/cleaved G-segment) as well as the crystal structures of the C-terminal domain of GyrA from *B. burgdorferi* (1SUU)^1^ (for C terminal domain of ParC) and N-terminal domain of GyrB from *E. coli* (for N-terminal domain of ParE)^2^. Unbound G-segment and the transported T-segment were generated using WinCoot^3^. N-terminal domain of ParC is shown in blue, C-terminal domain of ParC in cyan, N-terminal domain of ParE in purple, C-terminal domain of ParE in yellow, G-segment in green and T-segment in red.
(8.24 MB MPG)

## References

[1] Schoeffler AJ, Berger JM (2008). DNA topoisomerases: harnessing and constraining energy to govern chromosome topology.. Q Rev Biophys.

[2] Nitiss JL (2009). Targeting DNA topoisomerase II in cancer chemotherapy.. Nat Rev Cancer.

[3] Drlica K, Malik M, Kerns RJ, Zhao X (2008). Quinolone-mediated bacterial death.. Antimicrob Agents Chemother.

[4] Sissi C, Palumbo M (2009). Effects of magnesium and related divalent metal ions in topoisomerase structure and function.. Nucleic Acids Res.

[5] Laponogov I, Sohi MK, Veselkov DA, Pan XS, Sawhney R (2009). Structural insight into the quinolone-DNA cleavage complex of type IIA topoisomerases.. Nat Struct Mol Biol.

[6] Ellsworth EL, Tran TP, Showalter HD, Sanchez JP, Watson BM (2006). 3-aminoquinazolinediones as a new class of antibacterial agents demonstrating excellent antibacterial activity against wild-type and multidrug resistant organisms.. J Med Chem.

[7] Tran TP, Ellsworth EL, Sanchez JP, Watson BM, Stier MA (2007). Structure-activity relationships of 3-aminoquinazolinediones, a new class of bacterial type-2 topoisomerase (DNA gyrase and topo IV) inhibitors.. Bioorg Med Chem Lett.

[8] Huband MD, Cohen MA, Zurack M, Hanna DL, Skerlos LA (2007). In vitro and in vivo activities of PD 0305970 and PD 0326448, new bacterial gyrase/topoisomerase inhibitors with potent antibacterial activities versus multidrug-resistant gram-positive and fastidious organism groups.. Antimicrob Agents Chemother.

[9] Pan XS, Gould KA, Fisher LM (2009). Probing the differential interactions of quinazolinedione PD 0305970 and quinolones with gyrase and topoisomerase IV.. Antimicrob Agents Chemother.

[10] Pan XS, Ambler J, Mehtar S, Fisher LM (1996). Involvement of topoisomerase IV and DNA gyrase as ciprofloxacin targets in *Streptococcus pneumoniae*.. Antimicrob Agents Chemother.

[11] Pan XS, Fisher LM (1997). Targeting of DNA gyrase in *Streptococcus pneumoniae* by sparfloxacin: selective targeting of gyrase or topoisomerase IV by quinolones.. Antimicrob Agents Chemother.

[12] Leo E, Gould KA, Pan XS, Capranico G, Sanderson MR (2005). Novel symmetric and asymmetric DNA scission determinants for *Streptococcus pneumoniae* topoisomerase IV and gyrase are clustered at the DNA breakage site.. J Biol Chem.

[13] Dong KC, Berger JM (2007). Structural basis for gate-DNA recognition and bending by type IIA topoisomerases.. Nature.

[14] West KL, Meczes EL, Thorn R, Turnbull RM, Marshall R (2000). Mutagenesis of E477 or K505 in the B' domain of human topoisomerase IIβ increases the requirement for magnesium ions during strand passage.. Biochemistry.

[15] Noble CG, Maxwell A (2002). The role of GyrB in the DNA cleavage-religation reaction of DNA gyrase: a proposed two metal-ion mechanism.. J Mol Biol.

[16] Deweese JE, Burgin AB, Osheroff N (2008). Human topoisomerase IIα uses a two-metal-ion mechanism for DNA cleavage.. Nucleic Acids Res.

[17] Lu XJ, Olson WK (2003). 3DNA: a software package for the analysis, rebuilding and visualization of three-dimensional nucleic acid structures.. Nucleic Acids Res.

[18] Lu XJ, Olson WK (2008). 3DNA: a versatile, integrated software system for the analysis, rebuilding and visualization of three-dimensional nucleic-acid structures.. Nat Protoc.

[19] Laponogov I, Veselkov DA, Sohi MK, Pan XS, Achari A (2007). Breakage-reunion domain of *Streptococcus pneumoniae* topoisomerase IV: crystal structure of a gram-positive quinolone target.. PLoS One.

[20] McPherson A, Cudney B (2006). Searching for silver bullets: an alternative strategy for crystallizing macromolecules.. J Struct Biol.

[21] Otwinowski Z, Minor W (1997). Processing of X-ray diffraction data collected in oscillation mode.. Method Enzymol.

[22] McCoy AJ, Grosse-Kunstleve RW, Adams PD, Winn MD, Storoni LC (2007). Phaser crystallographic software.. J Appl Crystallogr.

[23] Adams PD, Grosse-Kunstleve RW, Hung LW, Ioerger TR, McCoy AJ (2002). PHENIX: building new software for automated crystallographic structure determination.. Acta Crystallogr D Biol Crystallogr.

[24] Emsley P, Cowtan K (2004). Coot: model-building tools for molecular graphics.. Acta Crystallogr D Biol Crystallogr.

[25] Morris AL, MacArthur MW, Hutchinson EG, Thornton JM (1992). Stereochemical quality of protein structure coordinates.. Proteins.

[26] Laskowski RA, MacArthur MW, Moss DS, Thornton JM (1993). PROCHECK: a program to check the stereochemical quality of protein structures.. J Appl Crystallogr.

[27] DeLano WL (2008). The PyMOL molecular graphics system.. http://www.pymol.org.

