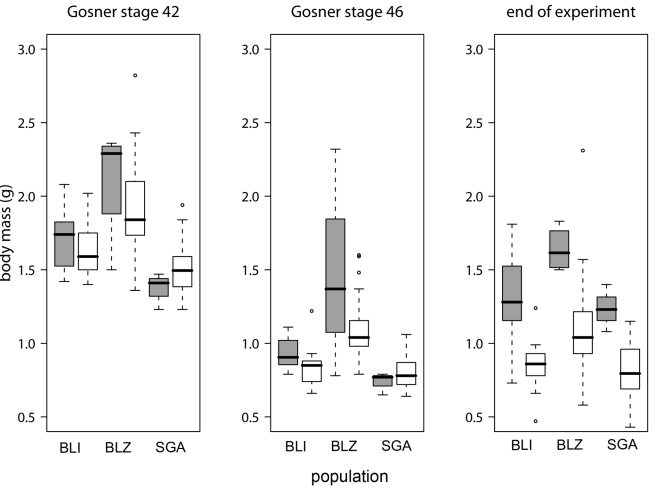# Correction: Structural Basis of Gate-DNA Breakage and Resealing by Type II Topoisomerases

**DOI:** 10.1371/annotation/33d82b59-59a3-4412-9853-e78e49af76b9

**Published:** 2010-07-08

**Authors:** Ivan Laponogov, Xiao-Su Pan, Dennis A. Veselkov, Katherine E. McAuley, L. Mark Fisher, Mark R. Sanderson

Figure 2 panel B contains an error. Please view the correct figure here: 

**Figure:**