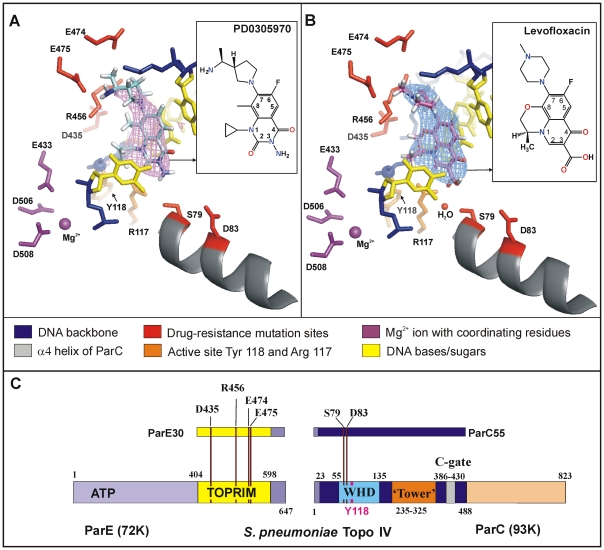# Correction: Structural Basis of Gate-DNA Breakage and Resealing by Type II Topoisomerases

**DOI:** 10.1371/annotation/deacc2fd-665b-4736-b668-dc69a38bb4f9

**Published:** 2010-07-09

**Authors:** Ivan Laponogov, Xiao-Su Pan, Dennis A. Veselkov, Katherine E. McAuley, L. Mark Fisher, Mark R. Sanderson

There was an error in Figure 2. Please view the correct figure here: 

**Figure:**