# Supplementary figures and images for: Structural Basis of Gate-DNA Breakage and Resealing by Type II Topoisomerases
Source: PLoS One. 2010 Jun 28;5(6):e11338. doi: 10.1371/journal.pone.0011338 (PMC2893164; doi:10.1371/journal.pone.0011338)

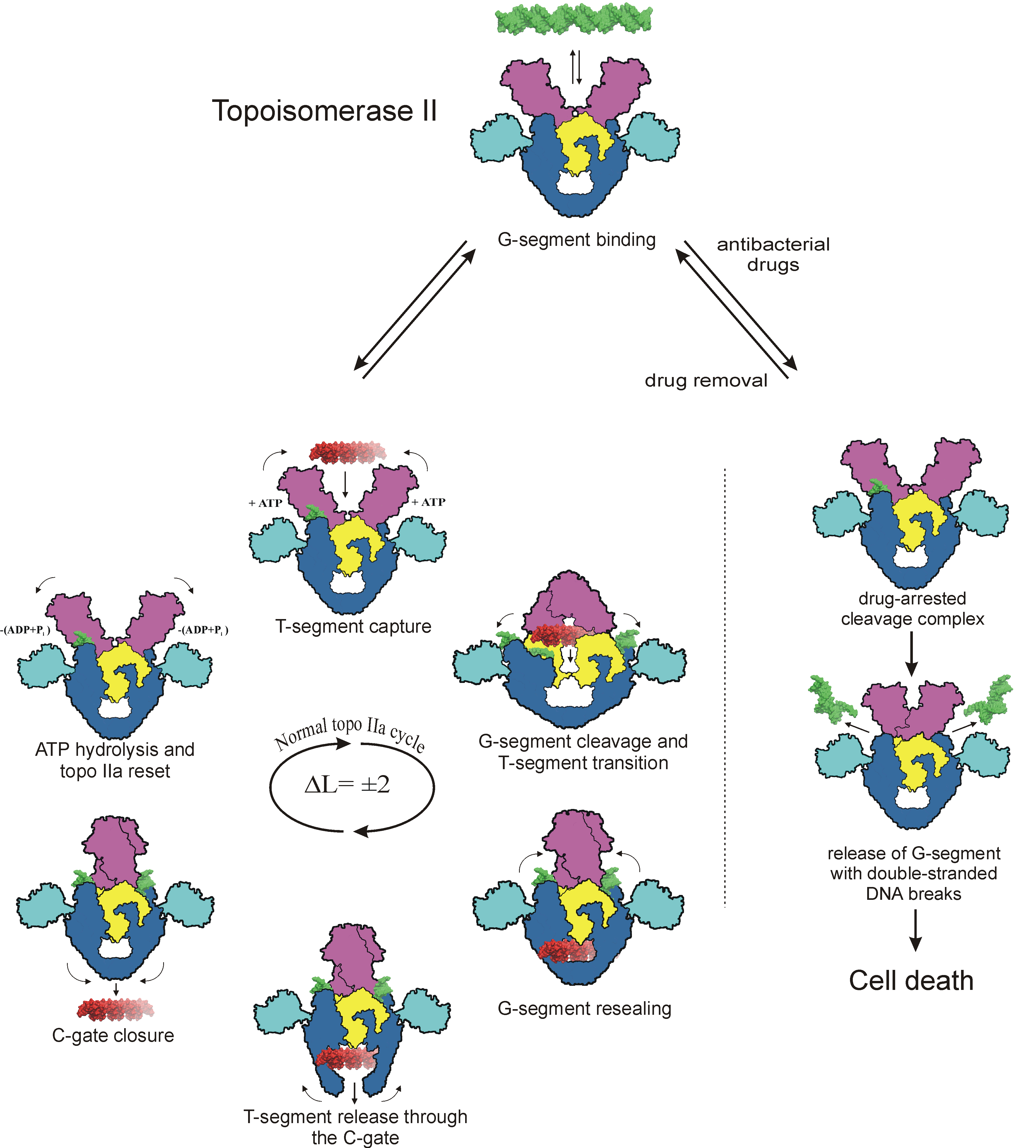

Supplement: Figure S1 — Schematic model of the DNA transport mechanism employed by a type II DNA topoisomerase and its inhibition by antibacterial/anticancer drug action. The diagram is built using a model of the full-length topo IV which, in turn, is based on the crystal structure of the drug-free cleavage complex of topo IV from S. pneumoniae with cleaved DNA (reported in this paper, 3KSA, covering the N-terminal domain of ParC, C-terminal domain of ParE and bound/cleaved G-segment) as well as the crystal structures of the C-terminal domain of GyrA from B. burgdorferi (1SUU)1 (for C-terminal domain of ParC) and the N-terminal domain of GyrB from E. coli (1EI1) (for N-terminal domain of ParE)2. Unbound G-segment and the transported T-segment were generated using WinCoot3. N-terminal domain of ParC is shown in blue, C-terminal domain of ParC is in cyan, N-terminal domain of ParE is in purple, C-terminal domain of ParE is in yellow, G-segment is in green and T-segment is in red. ΔL stands for a change in the linking number of the DNA per cycle. An animated version of this scheme is available as the Supporting Movie S1 online. (2.41 MB TIF) [file pone.0011338.s001.tif]

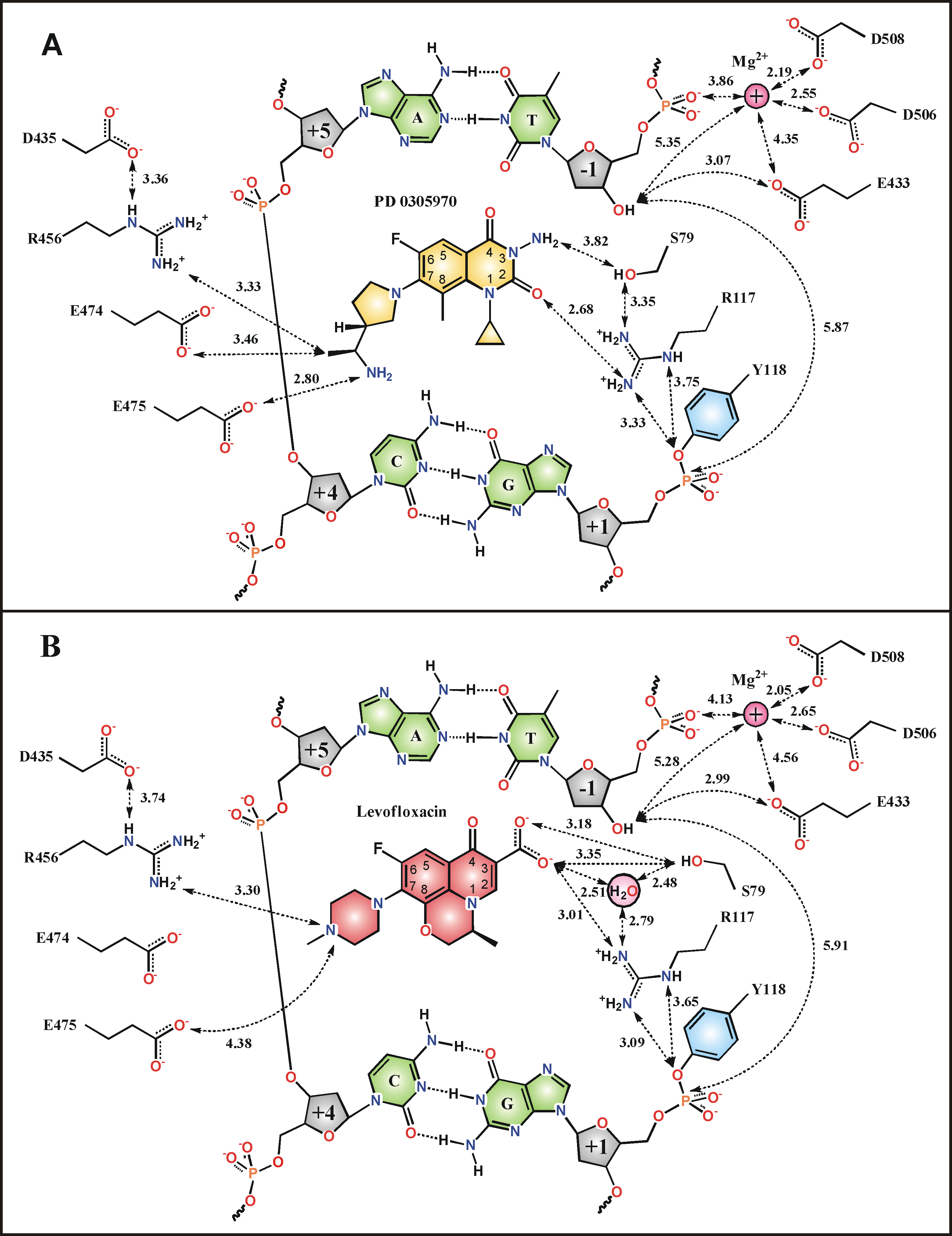

Supplement: Figure S2 — Key interactions within drug-stabilized cleavage complexes of S. pneumoniae topoisomerase IV. The cleavage complexes are stabilized by quinazolinedione PD 0305970 (A) and quinolone levofloxacin (B). The important inter-atomic distances are shown in Ångstroms and indicated by arrows. Magnesium ion is in purple, tyrosine is in cyan, DNA bases are in green, DNA sugars are in grey, PD 0305970 is in yellow, levofloxacin is in ‘rose’ and water molecule is in light pink. Nitrogen, oxygen and phosphorus atoms are in blue, red and orange, respectively. (1.15 MB TIF) [file pone.0011338.s002.tif]

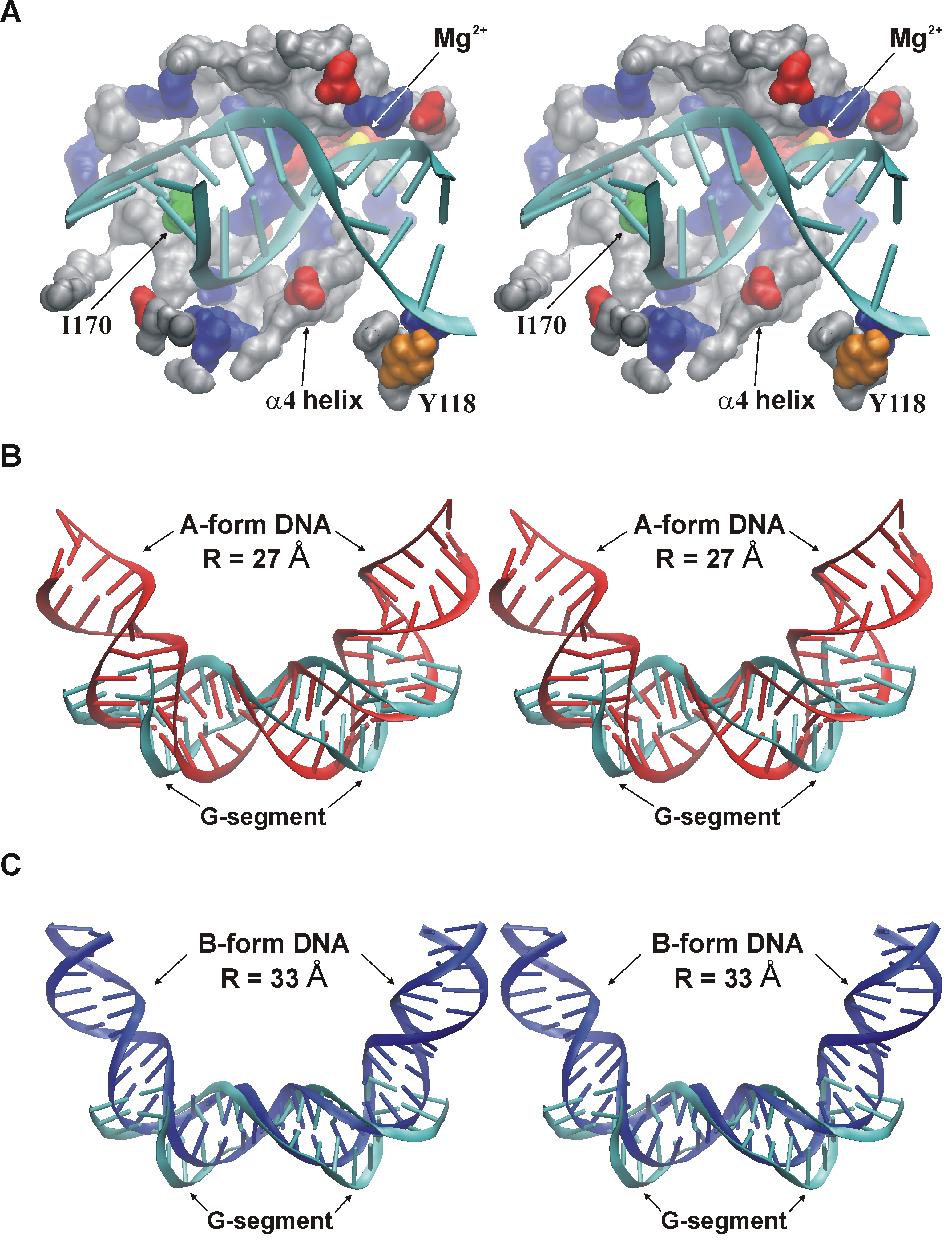

Supplement: Figure S3 — Specifics of the G-segment binding groove and DNA curvature (stereograms). A, Key elements of the DNA-binding cleft of topo IV from S. pneumoniae. Residues within 5 Å distance from the DNA molecule are shown in surface representation. Side-chains of basic/acidic amino acids are in blue/red respectively. Active site tyrosine is in orange, the side-chain of intercalating isoleucine 170 is in green, the magnesium ion is in yellow. The G DNA fragment is shown in cartoon representation (cyan). The rest of the complex is omitted for clarity. B and C, modelled ideal A-form and B-form DNA molecules with curvatures of 27 and 33 Å respectively superposed onto the re-sealed G-segment. Models were prepared using CNS4. The figure panels were rendered in VMD5. (4.38 MB TIF) [file pone.0011338.s003.tif]
