# Supplementary material for: Structural Basis of Gate-DNA Breakage and Resealing by Type II Topoisomerases
Source: PLoS One. 2010 Jun 28;5(6):e11338. doi: 10.1371/journal.pone.0011338 (PMC2893164; doi:10.1371/journal.pone.0011338)
Supplement: Table S1 — Data collection and refinement statistics and Supporting References. (0.06 MB DOC) [file pone.0011338.s004.doc]

**Table S**1. Data collection and refinement statistics.

|  | Native complex with PD 0305970 | Levofloxacin-soaked complex | EDTA crystal complex (Cleaved drug-free complex) | EDTA-Mg crystal complex (Re-sealed drug-free complex) |
| --- | --- | --- | --- | --- |
| **Data collection** |  |  |  |  |
| Space group | P3_2_ | P3_2_ | P3_2_ | P3_2_ |
| Cell dimensions |  |  |  |  |
| *a*, *b*, *c* (Å) | 122.51, 122.51, 178.30 | 122.41, 122.41, 178.29 | 116.91, 116.91, 183.76 | 116.76, 116.76, 182.81 |
| *α, β, γ* (°) | 90,90,120 | 90,90,120 | 90,90,120 | 90,90,120 |
| Resolution (Å) * | 3.10-50.00 (3.10-3.21) | 2.90-50.00 (2.90-3.00) | 3.30-50.00 (3.30-3.42) | 3.50-50.00 (3.50-3.63) |
| *R*_sym_* or *R*_merge_* | 0.075 (0.408) | 0.074 (0.422) | 0.080 (0.461) | 0.065 (0.400) |
| *I*/σ*I* * | 17.16 (3.563) | 15.20 (2.19) | 15.73 (3.51) | 21.96 (4.33) |
| Completeness (%)* | 99.2 (100) | 98.1 (100) | 98.0 (100) | 97.7 (100) |
| Redundancy* | 5.8 (5.8) | 5.6 (5.8) | 5.7 (5.7) | 6.1 (6.5) |
|  |  |  |  |  |
| **Refinement** |  |  |  |  |
| Resolution (Å) | 3.10-34.13 | 2.90-26.73 | 3.30-29.40 | 3.50-29.32 |
| No. reflections | 53839 | 64564 | 41250 | 34220 |
| *R*_work/_ *R*_free_ (%) | 18.6/22.8 | 18.3/21.8 | 17.6/21.8 | 18.1/22.6 |
| No. atoms |  |  |  |  |
| Protein | 10282 | 10721 | 10079 | 9865 |
| DNA | 730 | 730 | 730 | 730 |
| Ligand/ion | 102 | 94 | 2 | 2 |
| Water | 5 | 14 | 7 | 4 |
| B-factors** |  |  |  |  |
| Protein | 112.16 | 88.43 | 137.02 | 148.15 |
| DNA | 115.76 | 89.33 | 130.48 | 141.99 |
| Ligand/ion | 137.61 | 112.96 | 130.35 | 171.46 |
| Water | 86.26 | 73.63 | 119.60 | 120.35 |
| R.m.s deviations |  |  |  |  |
| Bond lengths (Å) | 0.025 | 0.010 | 0.027 | 0.027 |
| Bond angles (º) | 1.308 | 1.403 | 1.295 | 1.441 |

*Highest resolution shell is shown in parenthesis.

**Refined in Phenix using TLS (see Methods section for details).

**Supporting References**

1. Corbett KD, Shultzaberger RK, Berger JM (2004) The C-terminal domain of DNA gyrase A adopts a DNA-bending β-pinwheel fold. Proc Natl Acad Sci USA 101: 7293-7298.
2. Brino L, Urzhumtsev A, Mousli M, Bronner C, Mitschler A et al. (2000) Dimerization of Escherichia coli DNA-gyrase B provides a structural mechanism for activating the ATPase catalytic center. J Biol Chem 275: 9468-9475.

3. Emsley P, Cowtan K (2004) Coot: model-building tools for molecular graphics. Acta Crystallogr D Biol Crystallogr 60: 2126-2132.

4. Brünger AT, Adams PD, Clore GM, Gros P, Grosse-Kunstleve RW et al. (1998) Crystallography & NMR System (CNS), A new software suite for macromolecular structure determination. Acta Crystallogr D Biol Crystallogr 54: 905-921.

5. Humphrey W, Dalke A, Schulten K (1996) VMD - Visual Molecular Dynamics. J Molec Graphics 14: 33-38.
